# Differences in Inhibitory Control between Impulsive and Premeditated Aggression in Juvenile Inmates

**DOI:** 10.3389/fnhum.2017.00373

**Published:** 2017-07-24

**Authors:** Zhuo Zhang, Qianglong Wang, Xu Liu, Ping Song, Bo Yang

**Affiliations:** ^1^School of Sociology, China University of Political Science and Law Beijing, China; ^2^School of Criminal Justice, China University of Political Science and Law Beijing, China; ^3^Criminal Investigation College, Criminal Investigation Police University of China Shenyang, China

**Keywords:** violence, impulsive aggression, premeditated aggression, event-related potentials, inhibitory control

## Abstract

Inhibitory control dysfunction was considered a universal characteristic of violent offenders. The aim of this study was to examine differences in inhibitory control between two subtypes of violent youth; those displaying predominantly impulsive and those presenting predominantly premeditated aggression (PM). Forty-four juvenile offenders, defined on the basis of the Procedures for the Classification of Aggressive/Violent Acts (Stanford and Barratt, 2001) participated (*N* = 23: impulsive; *N* = 21 premeditated). A visual Go/NoGo task was used to compare behavioral responses and event-related potentials (ERPs) between groups. The task contained two letters (W and M), W was the Go stimulus and M the NoGo stimulus. The impulsive youth showed a significantly greater decrease in N2 latency for Go relative to NoGo trials than the premeditated aggressive youth. The differentiation in N2 amplitude between Go and NoGo (N2d) was negatively correlated with impulsivity of aggression. Both groups showed no significant central NoGo P3. Our findings suggest that impulsive violent youth show stronger prepotent responses and impaired conflict monitoring during early inhibitory control processing relative to premeditated aggressive youth. Both impulsive and premeditated violent youth may show impaired response inhibition at the late processing stage of inhibitory control.

## Introduction

Aggressive behavior in both children and adults can be classified into two distinct subtypes, impulsive and premeditated aggression (PM; Dodge and Coie, 1987; Barratt, 1991). Impulsive aggression (IA; also known as reactive, affective or defensive aggression) is more characterized by spontaneous, emotion-driven response to perceived threat or provocation. PM (also known as instrumental, predatory or proactive aggression), on the other hand, is more characterized by deliberate, non-emotional behavior to obtain a specified goal or intimidate others (Crick and Dodge, 1996). Most individuals display both IA and PM to varying degrees and the two subtypes tend to be highly correlated (Poulin and Boivin, 2000; Kempes et al., 2006). However, one form is usually predominant. Although the meaningfulness of the dichotomy has been questioned (Bushman and Anderson, 2001), increasing evidence suggests that the two subtypes of aggression display differences in familial precursors, behavioral outcomes, social-cognitive and emotional processes and social experiences (Dodge et al., 1997; Raine et al., 2006; Hubbard et al., 2010).

Self-reported impulsivity has been shown to be positively correlated with levels of IA, but not with PM in both clinical and non-clinical samples (Barratt and Felthous, 2003; Ramírez and Andreu, 2006). Executive dysfunction (Giancola et al., 1996; LeMarquand et al., 1998), in particular poor inhibitory control, has been associated with impulsivity and IA (Buss and Plomin, 1975; Barratt, 1991; Vigil-Colet and Codorniu-Raga, 2004). In contrast, PM has been shown to be significantly correlated with level of psychopathic traits (i.e., emotion dysfunction (reduced guilt and empathy) and antisocial behavior; Murrie et al., 2004; Raine et al., 2006; Flight and Forth, 2007). Young adults who committed more premeditated violence scored higher in psychopathic traits compared to those with primarily histories of impulsive violence (Kruh et al., 2005). Psychopathic traits have been less consistently associated with executive control/disinhibition (Morgan and Lilienfeld, 2000).

Relatively few studies have attempted to distinguish between impulsive and premeditated violent offenders using neurophysiological measures of inhibitory control (Stanford et al., 2003). The Go/NoGo task has been widely used to measure inhibitory control. In this task, participants are required to execute a prepared response to Go stimuli and suppress the response to NoGo stimuli. Two event-related potential (ERP) components, the N2 and P3, are elicited in Go/NoGo tasks. In particular, N2 is significantly greater for NoGo relative to Go trials with a maximum amplitude over frontal locations at 150–400 ms after stimulus onset, the so-called NoGo N2 effect (Falkenstein et al., 1999). Moreover, P3 is also significantly greater for NoGo relative to Go trials with a maximum amplitude over frontal locations at 300–600 ms after stimulus onset, the so-called NoGo P3 effect or NoGo P3 “anteriorization” (Kopp et al., 1996; Falkenstein et al., 1999). There have been suggestions that the NoGo N2 effect reflects top-down inhibition suppressing prepotent responses (Falkenstein et al., 1999) or conflict monitoring processing before a correct response (Nieuwenhuis et al., 2003; Donkers and van Boxtel, 2004). The NoGo P3 effect has been hypothesized to reflect either the response inhibition on the NoGo task or the contribution of movement production process on the Go task (Bruin and Wijers, 2002; Salisbury et al., 2004; Smith et al., 2008).

Relatively few ERP studies of violent offenders have been conducted. Chen et al. (2005) observed decreased N2 but not P3 amplitudes in impulsive violent offenders compared to normal non-offender controls. Several studies have used ERP with individuals with psychopathy. Kiehl et al. (2000) reported that the NoGo N2 effect was absent in psychopaths while the Go P3 was larger at anterior sites than the NoGo P3 in contrast to non-psychopathic offenders. However, Munro et al. (2007) found no differences in frontal NoGo N2 and P3 effect between violent offenders and comparison individuals as a function of psychopathy. Moreover, to our knowledge, no studies have examined ERP differences between impulsive and premeditated offenders in the same study.

The goal of the current study was to examine NoGo N2 and P3 effects in impulsive and premeditated violent offenders using a visual Go/NoGo paradigm. On the basis of the previous literature, we predicted that the N2-Nogo effect of impulsive offenders was less than that of premeditated offenders (longer latencies and smaller amplitude), and the P3-Nogo effect of impulsive offenders was larger than premeditated offenders (shorter latencies and larger amplitude). Impulsive offenders would show reduced inhibitory control relative to premeditated aggressive individuals.

## Materials and Methods

### Participants

The participants were 44 male juvenile offenders (age 19.45 ± 1.15 at the time of testing; years of education 8.69 ± 1.84) selected from a sample of 172 inmates at a prison for juvenile delinquents. For all inmates, an initial self-reported questionnaire was conducted to collect the criminal history and demographic data. The inclusion criteria were: (1) violent offenders who committed murder, robbery, assault and rape; (2) age 14 through 18 years at the time of committing crime, and 16 through 21 years at the time of testing; (3) education level above sixth grade; and (4) normal or corrected-to-normal visual acuity. The study was approved by the Prison Administration Bureau of the Ministry of Justice. All participants, and for minors also both parents/caregivers, gave written informed consent. This study was approved by the Ethical Committee of China University of Political Science and Law in accordance with the ethical principles of Declaration of Helsinki.

### Procedure

Procedures for the Classification of Aggressive/Violent Acts developed by Stanford were adopted (Stanford and Barratt, 2001). Institutional file data—consisting of case histories, criminal records, medical and psychiatric data and daily summaries of institutional behavior—were used to classify the aggressive behavior of each offender. Twenty-one inmates were excluded for any of the following criteria: (1) a verbal IQ below 80 on the Wechsler Intelligence Scale for Children-III (WISC-III); (2) current diagnosis of a DSM-IV-TR Axis I psychiatric disorder assessed with the Structured Clinical Interview for DSM-IV-TR Axis I Disorders, Research Version, Non-patient Edition (SCID-I/NP); (3) present use of medication; (4) a neurological or seizure disorder; and (5) a self-reported history of head injury. Thus, the remaining 120 inmates were interviewed to evaluate the aggressor’s motivation and behavioral control during aggressive acts by four trained interviewers. The Impulsive/Premeditated Aggression Scale (IPAS) was then administered to identify the predominant type of violent acts. After completing each step of formal records review, the semi-structured aggression interview and the IPAS, the interviewers initially classified inmate’s aggressive behavior as premeditated, impulsive or can’t classify. The overall classification of aggressive acts were then discussed and determined by consensus of the research team (authors ZZ, QW, XL, PS and BY). Those could not be classified due to inconsistent diagnosis or insufficient information was excluded from further study.

This lead to the identification of 44 inmates: half who were classified as impulsive and half who were classified as premeditated violent offenders who met the inclusion criteria and agreed to participate. However, five participants were dropped from further analyses: one for technical issues in data recording, one for dropout during recording, and three for absence. There were no significant differences in age and education level between the remaining 23 impulsive and 21 premeditated aggressive participants (age: impulsive group = (19.46 ± 1.16) years, premeditated group = (19.13 ± 1.49) years; education: impulsive group = (8.69 ± 1.84) years, premeditated group = (8.58 ± 1.24) years, *t*-test *p* > 0.05). Regarding type of crime committed, 16 (36.4%, nine from the IA group and seven from the PM group) inmates were convicted for robbery, seven (15.9%, 4 IA and 3 PM) for assault, seven (15.9%, 4 IA and 3 PM) for rape, five (11.3%, 3 IA and 2 PM) for gang fight, and one (2.3%, PM) for murder. The other eight (18.1%) had committed multiple offences, of which three (6.8%, 1 IA and 1 PM) were convicted for assault and rape, three (6.8%, PM) for robbery and theft, and two (4.5%, PM) for rape and robbery. There were no significant differences in criminal types between impulsive and premeditated groups (Fisher’s exact test, *p* > 0.05).

### The Stanford and Barratt (2001) Semi-Structured Aggression Interview

The semi-structured aggression interview was used to help gather data beyond the review of formal records. Inmates who showed inconsistency between the formal records and self-reported information in the interview were excluded. To evaluate the aggressor’s motivation and behavioral control during aggressive acts, 11 items were included to answer two main questions: (1) is there evidence for a motive that was the basis for the aggressive act (e.g., “Did you plan ahead of time to commit the aggressive act? If yes, how long before the aggressive act did you plan?”); and (2) is there evidence that the provoking stimulus resulted in the aggressor impulsively responding (e.g., “Were you agitated or out of control during the aggressive act? If yes, how did you feel (e.g., angry, depressed, jealous)?”). In their original study, Stanford and Barratt reported an internal consistency reliability of 0.91 and a kappa of 0.83, with male aggressors assessed by two independent interviewers.

### Impulsive/Premeditated Aggression Scale (IPAS)

The IPAS is a 30-item self-report instrument designed to characterize aggressive behavior as predominately impulsive or predominately premeditated in nature. Items are scored on a 5-point Likert-type scale (Strongly Disagree, Disagree, Neutral, Agree and Strongly Agree) and 20 items are scored to yield two subscale scores: IA and PM. The IPAS has been validated for juveniles, and its reliability estimates range from 0.72 to 0.82 (Stanford et al., 2003; Kockler et al., 2006; Mathias et al., 2007). The categorical scoring technique was used to classify participants into impulsive and premeditated groups. Items for which the individual answers Strongly Agree or Agree were rated as positive. The total number of positive items was determined for both the IA and PM scales. Then the percentage of positive items for IA and PM scales were calculated respectively. Difference between IA and PM percentage (*P*_d_) was used to classify participants into predominately impulsive or predominately premeditated subtypes of aggression. Inmates with *P*_d_ in the lowest 25% were then excluded.

### Stimuli and Procedure

**Figure 1:**
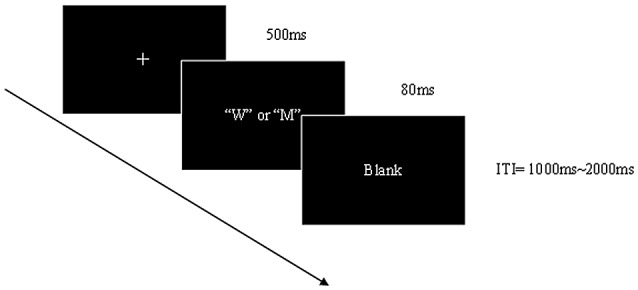
Sequence of events in Experiment.

The stimuli were single black capital letters (W and M) of the same size (8 cm * 8 cm) in gray background (Figure 1). For half of the participants, W was the Go stimulus and M the NoGo stimulus. Each stimulus was presented in the center of a computer screen (light degree = 60 cd/m^2^) by the Neuroscan STIM-2 system for 80 ms. There was then a random inter-trial intervals (ITI) between 1000 ms and 2000 ms (mean = 1500 ms). The participants were required to respond by pressing a button as quickly as possible after the Go stimuli appeared and to withhold the response when the NoGo stimuli appeared. An equal probability Go/NoGo task was adopted to elicit robust NoGo components and eliminate the bias toward the prepotent event types. Before ERP recording, participants performed two practice blocks each consisting of 20 Go and NoGo trials. Following this, the participants received two experiment blocks each of which contained 50 Go and 50 NoGo stimuli.

The experiment took place in the psychological consulting room of the prison. Participants faced a monitor placed 100 cm from their eyes, with a visual angle of 5.7° × 4°. All participants were asked not to consume food for 3 h prior to testing. During the experiment, participants were instructed to watch the center of the screen, relax and minimize eye blinks or body movements.

### EEG Recording

Scalp voltages were recorded using a 32-channel Ag/AgCl electrodes cap (10–20 International System). The acquisition software was Neurolab^®^ digital amplifier. The electrodes were referenced to the nose and the ground electrode was on the forehead (frontal midline). Eye movements were monitored with a supraorbital vertical lead and a horizontal lead placed on the external canthus of the eyes. Electrode impedance was maintained below 5 KΩ. The EEG signals were recorded continuously with a bandpass of 0.05–100 Hz and digitized at a 1000 Hz sampling rate.

Ocular artifacts were corrected with an eye-movement correction algorithm described by Semlitsch et al. (1986). ERP epochs with a 1000 ms duration were extracted (including 200 ms before and 800 ms after stimulus onset) and corrected by the preceding 200 ms baseline. EEG exceeding ± 100 μV were automatically rejected as artifacts. Individual ERP averages were derived for correct Go and NoGo trials, and digitally low-pass filtered with zero phase shift (30 Hz, 24 dB/octave).

### Data Analysis

Behavioral indices, including Go response time, hit rate and false alarm rate, and the classical signal detection measures, response bias (log β) and discriminative sensitivity (d′) were calculated to allow group comparisons by *t*-test.

The N2 component was quantified as the most negative amplitude within a 150–300 ms window following stimulus onset. The P3 component was quantified as the most positive amplitude within 300–550 ms following stimulus onset. In order to highlight the NoGo effect, difference waves (NoGo minus Go), designated as N2d and P3d, were computed for N2 and P3 (Falkenstein et al., 1999). The peak latency was respectively computed at the recording sites: Fz and Fcz, for N2 and P3.

Repeated measures Analysis of Variance (ANOVA) was performed to test for the different effects of response inhibition between two groups. Within-Subjects factors were Task (Go, NoGo) and Site (Fz, Fcz, Cz, Cpz, Pz). Years of education was incorporated as a covariate when the amplitude and latency of N2 and P3 were assessed. Pearson’s correlations were computed to characterize relations among IPAS scores, behavioral performance (response time, log β and d′) and ERP characteristics (amplitudes and latencies of N2d and P3d). The Greenhouse–Geisser ε-correction was used for any repeated measures containing more than one degree of freedom in the numerator. The statistical significance was set at the *p* < 0.05 level. Statistical analysis was performed with the statistical package SPSS 13.0.

## Results

### IPAS Data

Confirming our selection of participant groups, the impulsive offenders showed a higher level of impulsive aggressive characteristics (*P*_d_ = 0.203, *t* = 4.245, *p* < 0.01) and premeditated ones showed a higher level of premeditated aggressive characteristics (*P*_d_ = −0.087, *t* = −1.562, *p* < 0.05) on the IPAS; see Table 1.

**Table 1:** Behavioral performance and Impulsive/Premeditated Aggression Scale (IPAS) Scores of impulsive and premeditated groups.

	Impulsive (*N* = 23)		Premeditated (*N* = 21)			
	Mean	SD	Mean	SD	*t*	*p*
Response time	349.81	56.44	384.89	43.02	−1.736	0.096
Hits (%)	96.85	6.012	99.00	1.48	−1.251	0.232
False alarms (%)	12.46	14.65	3.92	4.52	2.002	0.064
Log β	−2.34	1.47	−2.227	1.30	0.356	0.825
d′	3.31	1.15	3.16	0.97	−0.224	0.725
Impulsive scale
Positive items	4.15	1.99	4.00	2.89		
Percentage (%)	52.92	25.1	32.67	23.92	4.245	0.001**
Premeditated scale
Positive items	2.92	1.73	5.42	2.88		
Percentage (%)	36.46	21.62	45.14	23.96	−1.562	0.0147*

*p < 0.05; **p < 0.01.

### Behavioral Data

Response outcomes for the Go tasks of the two groups are presented in Table 1. The impulsive aggressive offenders showed marginally shorter Go-response times (*t* = −1.736, *p* = 0.096) and false alarms (*t* = 2.002, *p* = 0.064) than the premeditated group. There were no significant group differences in the proportion of hits, log βs and d′s for Go trials. There were no significant group differences in the number of correct rejections for the NoGo trials.

### ERP Data

**Figure 2:**
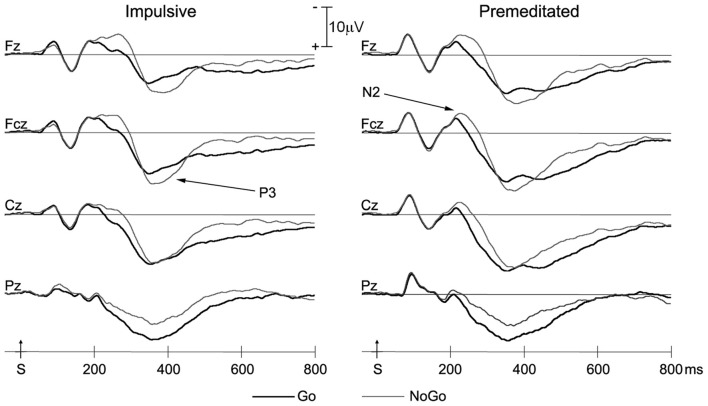
Grand averaged event-related potential (ERP) waveforms for Go and NoGo trials of impulsive and premeditated offenders at the four midline electrodes. Bold lines, Go trials; thin lines, NoGo trials; S, stimulus onset.

**Figure 3:**
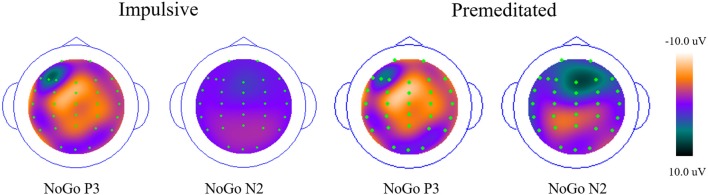
Topographic maps of ERP waveforms (P3 and N2) from the nogo condition across impulsive and premeditated aggression (PM).

Grand averaged ERP responses of impulsive and premeditated groups are shown in Figure 2. The N2 and P3, the two ERP components of interest, were measured separately for the Go and No Go trials for each participant. Topographic maps of N2 and P3 are shown in Figure 3.

#### N2 Component

As can be seen in Figure 2, the N2 in Go and NoGo trials for both groups was at maximum amplitude approximately 200 ms after stimulus onset. A 2 (group: Impulsive vs. Premeditated) × 2 (trial type: Go vs. NoGo) × 4 (electrode site) repeated measures ANOVA was conducted on the N2 amplitudes. This revealed a significant main effect for trial types: N2 amplitudes were greater for NoGo trials than Go trials (NoGo N2 = −7.27 ± 4.44 μV, Go N2 = −5.45 ± 3.09 μV, *F* = 14.12, *p* < 0.01, *η*^2^ = 0.38). There was also a main effect of electrode sites (*F* = 11.41, *p* < 0.01, *η*^2^ = 0.33). In both Go and Nogo trials, two groups showed significant larger N2 at Fz and Fcz than at other electrode sites (*p* < 0.05 in all cases). A 2 (group: Impulsive vs. Premeditated) × 2 (trial type: Go vs. NoGo) × 4 (electrode site) repeated measures ANOVA was conducted on the N2 latency. While there was no significant main effect for group, there was a significant group-by-trial type interaction. The impulsively aggressive youth showed a significantly greater increase in N2 latency for NoGo relative to Go trials relative to the premeditated aggressive youth (impulsive group: NoGo N2 = 229.92 ± 38.154 ms, Go N2 = 208.15 ± 35.66 ms; premeditated group: NoGo N2 = 226.6667 ± 30.107 ms, Go N2 = 221.4167 ± 26.593 ms; *F* = 5.19, *p* < 0.05, *η*^2^ = 0.184).

#### P3 Component

Both impulsive and premeditated offenders showed more NoGo P3 positivity at Fz and Fcz (*p* < 0.05), but not at Cz. The main effect of site for P3 amplitude was highly significant (*F* = 12.28, *p* < 0.01, *η*^2^ = 0.35), but no group or response main effect on the amplitude of P3 were found (all *p* > 0.05). The interaction between response and site was highly significant (*F* = 17.45, *p* < 0.01, *η*^2^ = 0.43). In both groups, the Go P3 peaked at Cz and the NoGo P3 peaked at Fcz. There was no group difference or interaction for P3 latency. No significant difference in P3 latency between Go and NoGo trials emerged for both groups (impulsive group: Go P3 = 369.46 ± 76.38 ms, NoGo P3 = 381.39 ± 42.98 ms, *F* = 0.41, *p* = 0.53, *η*^2^ = 0.03; premeditated group: 375.67 ± 56.42 ms, NoGo P3 = 389.17 ± 43.14 ms, *F* = 2.31, *p* = 0.16, *η*^2^ = 0.17).

### Correlation Analysis

The dimensional scores of IPAS, the IA and PM score, were calculated to represent an individual’s level of impulsive and premeditated aggressive behavior respectively (Stanford et al., 2003). In order to highlight the NoGo effect, difference waves (NoGo minus Go), designated as N2d and P3d, were calculated for each participant. The IA and PM are in fact partly overlapping with respect to their behavior, so partial correlations were used to assess the relationship between the IPAS dimensional score and the amplitude of N2d and P3d (Table 2). Significant negative correlation was observed between the impulsivity and N2d amplitude at Fz (partial *r* = −0.44, *p* < 0.05), which indicates NoGo N2 effect diminishes as the impulsiveness level increases. But the association between premeditation and the amplitude of N2d or P3d became negligible after controlling for scores on the IA subscale (*p* > 0.05). No other significant partial correlation was found between IPAS scores and ERP components.

**Table 2:** Partial correlation between average amplitude of N2d, P3d and aggressive behavior.

	Level of impulsive aggression	Level of premeditated aggression
N2d	
Fz	0.442 (*p* = 0.031*)	0.065 (*p* = 0.764)
Fcz	0.256 (*p* = 0.228)	0.210 (*p* = 0.324)
Cz	0.349 (*p* = 0.095)	0.126 (*p* = 0.556)
P3d	
Fz	−0.180 (*p* = 0.400)	0.065 (*p* = 0.763)
Fcz	−0.100 (*p* = 0.641)	−0.012 (*p* = 0.956)
Cz	0.009 (*p* = 0.966)	−0.055 (*p* = 0.799)

*p < 0.05 (two-tailed).

## Discussion

The present study is the first to explore differences in inhibitory control as indicated by ERP between impulsive and premeditated violent offenders. Behavioral data and two ERP components- N2 and P3 were examined in an equal probability visual Go/NoGo task. There were three main findings: first, the impulsively aggressive youth showed a significantly greater increase in N2 latency for NoGo relative to Go trials relative to the premeditated aggressive youth; Second, the differentiation in N2 amplitude between Go and NoGo trials (N2d) was negatively correlated with impulsivity of aggression; Third, the NoGo P3 effect was found in frontal but not central regions in both groups. These findings suggest that impulsive violent youth show stronger pre-potent responses and impaired conflict monitoring during early inhibitory control processing, and both impulsive and premeditated violent offenders might exhibit impaired inhibitory control but probably with different neurophysiological mechanisms.

The two subtypes of violent offenders did not differ significantly in behavioral performance. This may relate to the relative ease of the Go/NoGo task. Alternatively, it may reflect a power issue. It should be noted there were trends in the data such that the impulsive offenders showed shorter reaction times to targets and made more commission errors relative to the premeditated youth. These data are consistent with the idea that the impulsively aggressive youth were showing indications of impaired response inhibition. As such these data are at least tentatively in line with previous work indicating inhibitory control deficits in impulsively aggressive individuals (Chen et al., 2008; Feilhauer et al., 2012).

NoGo N2 and NoGo P3 in the Go/NoGo task are two primary components related to inhibitory control (Falkenstein et al., 1999). However, studies investigating the psychological significance of NoGo N2 and NoGo P3 in individuals showing high levels of aggression are sparse. Our results indicated that impulsive offenders showed both a significantly greater increase in N2 latency for NoGo relative to Go trials and a marginally significant reduction in N2 amplitude between Go and NoGo trials relative to the premeditated group. Notably, Chen et al. (2008) also reported inhibitory control deficits in impulsive violent offenders under the time pressure condition and a smaller N2d amplitude in impulsive violent offenders relative to matched controls (Chen et al., 2005). The NoGo N2 effect was traditionally presumed to be top-down inhibition (Falkenstein et al., 1999). But growing evidence supports the hypothesis that the N2 in Go/NoGo tasks reflects conflict arising from competition between the execution and the inhibition of a single response (Nieuwenhuis et al., 2003; Donkers and van Boxtel, 2004). The source of the N2 was localized to the anterior cingulate cortex (ACC; Bekker et al., 2005), which may play a key role in response conflict detection (Carter et al., 1998; Botvinick et al., 2001). The ACC has been found reliably activated in Go/NoGo responses, but the cognitive function of this activity is still controversial (Nieuwenhuis et al., 2003). According to the conflict detection hypothesis, the ACC serves to evaluate the demand for cognitive control by monitoring for occurrence of conflict in information processing (Braver et al., 2001). Our findings of significantly shorter latency in the Go condition than the Nogo condition suggests that impulsive violent offenders show a general tendency towards pre-potent responding. In addition, the slightly but non-significantly reduced N2d over the fronto-central area indicates that impulsive offenders might have impaired conflict monitoring and aberrant activation of the ACC during response inhibition. The inverse correlation between the NoGo N2 effect and the level of impulsivity in the juvenile violent offenders further supports this index as a measure of deficient response control.

In the current study, the impulsive violent offenders showed a relatively normal NoGo P3 effect. This is consistent with Chen et al. (2005) study where they also demonstrated no significant difference in the P3 component between impulsive violent offenders and matched controls. An fMRI study also found impulsiveness had no relationship with reduced activation in right orbital frontal cortex (OFC) and ventromedial prefrontal cortex in high risk people (Brown et al., 2015). The Go/NoGo P3 component in different groups showing issues with impulsiveness revealed inconsistent results. Thus, the NoGo P3 anteriorization effect were found in heroin addicts (Yang et al., 2009), smokers (Luijten et al., 2011) and ecstasy polydrug users (Gamma et al., 2005), but not in alcoholics (Kamarajan et al., 2005) or adult ADHD patients (Fisher et al., 2011). In healthy subjects, high impulsiveness scoring individuals were reported to exhibit significantly reduced NoGo P3 amplitudes compared to less impulsive individuals (Ruchsow et al., 2008). This inconsistency may reflect potential diverse neurobiological routes to higher levels of impulsiveness or specific aspects of the paradigms used across these studies and, in need, of empirical attention.

Some limitations of the present study should be noted. First, the present study did not include a non-violent control group. Future research may incorporate age-matched nonviolent imprisoned samples to further evaluate the results and extend their generalizability. Nevertheless, our findings shed light on the differences between youths with IA relative to PM. Second, due to the high correlations of IA and PM, most offenders especially those who committed multiple crimes were excluded and only 44 typical offenders were recruited in our experiment. The small sample size raises the possibility that the ERP differences are specific.

In conclusion, the present study provides neurophysiological evidence indicating that youths with IA and PM can be distinguished with respect to deficits in the mechanism of inhibitory control. Specifically, impulsive violent offenders appear to show particular difficulties in monitoring the competition between incompatible responses at an early stage of inhibitory control.

## Author Contributions

BY finished this article; all authors listed have made a substantial, direct and intellectual contribution to the work, and approved it for publication.

## Conflict of Interest Statement

The authors declare that the research was conducted in the absence of any commercial or financial relationships that could be construed as a potential conflict of interest.
